# Efficient Path Planning for a Microrobot Passing through Environments with Narrow Passages

**DOI:** 10.3390/mi13111935

**Published:** 2022-11-09

**Authors:** Cheng-Ming Huang, Shu-Hsien Hsu

**Affiliations:** Department of Electrical Engineering, National Taipei University of Technology, Taipei 106, Taiwan

**Keywords:** microrobot, rapidly exploring random tree, path planning

## Abstract

This paper presents an efficient path-planning algorithm for microrobots attempting to pass through environments with narrow passages. Because of the extremely small size of a microrobot, it is suitable for work in this kind of environment. The rapidly exploring random tree (RRT) algorithm, which uses random sampling points, can quickly explore an entire environment and generate a sub-optimal path for a robot to pass through it; however, the RRT algorithm, when used to plan a path for a microrobot passing through an environment with narrow passages, has the problem of being easily limited to local solutions when it confronts with a narrow passage and is unable to find the final path through it. In light of this, the objectives of the considered path planning problem involve detecting the narrow passages, leading the path toward an approaching narrow passage, passing through a narrow passage, and extending the path search more efficiently. A methodology was proposed based on the bidirectional RRT in which image processing is used to mark narrow passages and their entrances and exits so that the bidirectional RRT can be quickly guided to them and combined with the deterministic algorithm to find paths through them. We designed the methodology such that RRT generates the sampling points for path growth. The multiple importance sampling technique is incorporated with bidirectional RRT, named MIS-BiRRT, to make the path grow faster toward the target point and narrow passages while avoiding obstacles. The proposed algorithm also considers multiple candidate paths simultaneously to expand the search range and then retain the best one as a part of the planning path. After validation from simulation, the proposed algorithm was found to generate efficient path planning results for microrobots to pass through narrow passages.

## 1. Introduction

Due to the development of MEMS technology over the past decade, microrobots, which are constructed using small-sized sensors, actuators, processors, etc., have continued to be innovatively designed. Microrobots are characterized by extremely small size, light weight, low power consumption, and low cost [1]. Among microrobots, the origami robots in particular [2,3] have attracted much attention in recent years. The origami robots, which are usually structured with soft materials [4], could transform their shape to perform certain tasks which the rigid robots cannot. Therefore, they are especially suitable for entering complex and narrow spaces, such as those involved in vessel diagnosis of the human body [5,6], pipeline inspection of buildings [7,8], and military reconnaissance of otherwise inaccessible areas [9]. Most of the research on microrobots has only discussed the construction of their electromechanical systems and hardware mechanisms but has seldom explained how to navigate them to perform tasks in complex and narrow spaces. Hence, this paper focused specifically on designing the path-planning capability of a microrobot in order to efficiently pass through complex environments, especially those that contain narrow passages.

The field of path planning can be divided into two major methodologies: deterministic and stochastic. Common deterministic methodologies include Dijkstra’s algorithm [10], the artificial potential field algorithm [11], and the A* algorithm [12], which can design a fixed and unique path plan. The A* algorithm can find the definite and optimal path solution even if the search space is small, such as the kind of narrow passage considered in this paper. During the A* algorithm path search process, a point on the designed path is determined by calculating the distance between each point in the environment map and the starting/target point. These deterministic methods, however, are not suitable for path planning in vast and complex environments as they are computationally intensive and time-consuming. By contrast, the well-known stochastic path planning algorithms, such as probabilistic roadmap (PRM) [13] and rapidly exploring random tree (RRT) [14], have the characteristics of fast path searching and high variability. The RRT algorithm randomly samples a guiding point on the map and then generates a corresponding waypoint to repeatedly construct the path until it approaches the target point. Because this type of stochastic method is relatively fast, it can efficiently find a path in a wide-open space, unlike deterministic methods.

**Figure 1:**
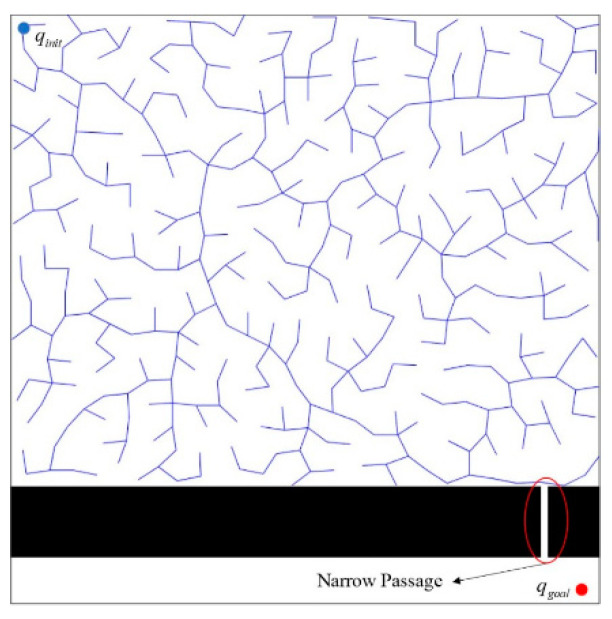
Traditional RRT algorithm experiencing difficulty trying to find a path through a narrow passage.

Due to the random sampling characteristic of RRT, several researches [15,16] have pointed out its inefficiency in planning a path through complicated environments and narrow passages. As illustrated in Figure 1, the probability of RRT generating waypoints in the narrow passage is reduced since the area of the narrow passage is small. If no waypoints are generated in a narrow passage, RRT cannot find a path through it. Some research [17,18,19,20,21] discussed the improvement of RRT path planning through narrow passages. Since the path planning under consideration here is that the map can be known in advance, marking the narrow passages first can quickly guide the RRT to find a pass through it. Most of them employed the bridge test method [19,22] to find the area of a narrow passage and mark specific waypoints around the narrow passage. The concept of the bridge test is to randomly sample a point that falls within an obstacle region. The adjacent obstacle can then be detected by sampling another point that is close to the previous sampling point within the region. Then, the middle of these two sampled points around a narrow passage is denoted as the specific waypoint to guide the RRT algorithm through it; however, detecting the adjacent obstacles around a narrow passage using this kind of sampling is slow and inefficient. The Locally Guided Multiple BiRRT* (LGM-BRRT*) [19], which constructs the local exploration with bidirectional RRT* between the adjacent identification points of narrow passages, would accelerate the path planning toward the narrow passages. Global sampling and local search trees [20] utilize image morphology to detect narrow passages, and in this paper, we proposed a path-planning concept in which the areas with narrow passages in them are first identified using image morphology instead of searching for a path in the entire space. Rather than blindly directing the RRT path planning toward a narrow passage even when there is another better path reaching the target point, we guided the RRT path growing toward a narrow passage only when the RRT path tree approached this narrow passage.

The related works of RRT improvement mentioned above can assist the RRT path in growing toward the narrow passages. These works could successfully plan a path passing through the rooms where only the entrance of each room is considered a narrow passage. However, when a narrow passage is thin and long, these related works may still hard find a path through this kind of narrow passage. Due to the characteristics of RRT uniform sampling, the search range easily affects the path-searching efficiency. The adaptive RRT [23] draws several points around a waypoint of the RRT tree, which is close to a narrow passage, to find the feasible extension of the RRT tree through the narrow passage. The principal component analysis could be utilized to sample the space around a narrow passage [18] for efficiently expanding the RRT tree through it. The concept of dynamic sampling space [15] is also proposed, where the growth of the RRT tree is concentrated within this sampling space to increase the probability of finding the exit of the narrow channel. Although the improvements in waypoint sampling could assist the RRT methodologies to plan a path through a narrow passage, the process of iterative sampling through a long narrow passage would be quite time-consuming. We guided the RRT algorithm to quickly generate such a path to a narrow passage and then used an effective method to deterministically plan a path through it. Since the A* algorithm generates good path-planning results in small and narrow regions, the path-planning algorithm employed here was switched to this kind of deterministic method for efficiently finding a path in the narrow passage.

In addition to the problem of crossing narrow passages, the path-searching process of traditional RRT methodology is often aimless due to its use of random sampling in complex environments. Even if the last generated waypoint is close to the target point, random sampling of the entire space could easily result in the next waypoint being far away from the target. This kind of aimless sampling during path searching makes path planning inefficient and consumes considerable time, especially in vast and complex environments. Therefore, several improvements were proposed for the waypoint sampling of RRT so that unnecessary searching and computational time can be reduced. The rapidly exploring random disjointed tree* (RRdT*) [24] establishes a Markov chain to consider the direction of path growth according to the growth of the previous waypoint. Peng et al. [25] utilized particle swarm optimization (PSO) to adjust waypoint sampling according to the path growth of RRT and apply it to the path planning of multi-UAVs. In addition, multi-RRT [26] incorporated a sampling mechanism named Goal Bias, which considers the position of the target point and accelerates the path growth of RRT toward the target point. Multi-RRT also draws multiple path growth directions simultaneously to increase the search range. Liu et al. [27] sampled new waypoints to avoid obstacles by detecting the obstacle edge when it was close to the growth path.

In this paper, we integrated the concepts of several waypoint sampling mechanisms to guide the path planning of a microrobot. Specifically, this paper proposes a multiple importance sampling bidirectional rapidly exploring random tree (MIS-BiRRT) to remedy the problem of RRT path planning through narrow passages that suffer from aimless path searching. The environmental map is first considered as an image for detecting the feature points of obstacles and narrow passages. The multiple importance sampling mechanism is used to draw the waypoints of a growing RRT path toward the target point, toward the entrance of a narrow passage, or so as to avoid obstacles. When the path grows into the entrance of a narrow passage, the RRT methodology switches to the deterministic path planning method to quickly obtain the optimal path through the narrow passage.

The rest of this paper is structured as follows. The path planning problem is first formulated, and the path planning plus image feature detection for narrow passages is explained in Section 2. Section 3 describes the multiple importance sampling mechanism and multiple new waypoints generation of the proposed MIS-BiRRT. The simulation results and comparisons are presented in Section 4 to verify the efficiency of the proposed MIS-BiRRT. Finally, the conclusions are given in Section 5.

## 2. Problem Formulation and Path Planning in Narrow Passages

**Figure 2:**
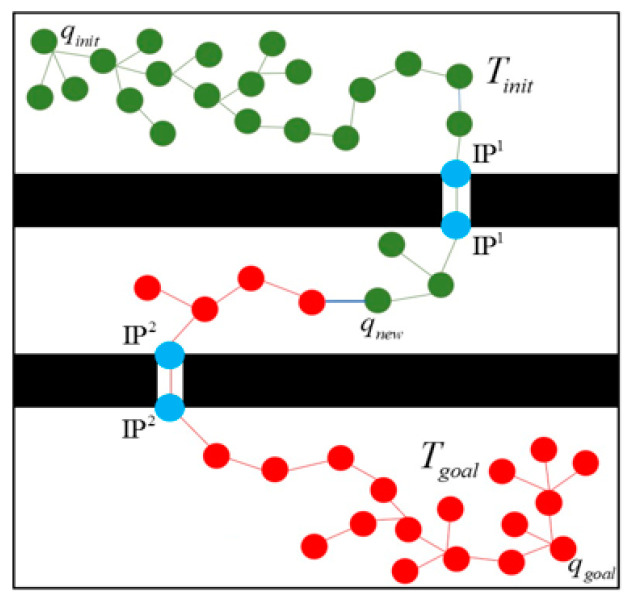
Path planning using different methodologies for narrow passages and for broad areas. (Blue: identification points of narrow passages; Green and red: growing waypoints of BiRRT in the broad areas).

A two-dimensional environment map E, which is assumed to be known in advance, contains the free-moving space F and obstacle space O. The goal of the entire path-planning process of a microrobot is to find a collision-free path *τ* that can lead the microrobot from the starting point $q_{init}$ to the target point $q_{goal}$ in F. *E* may also contain several unlabeled narrow passages P through which a microrobot of size *r* could pass, i.e., P∈F. Planning path *τ* using just the RRT algorithm would be difficult because of the small search area of the narrow passage. Since the map can be known in advance, marking the narrow passages can quickly guide the RRT to pass through them. The objectives of the considered path planning problem can be defined as detecting the narrow passages, leading the path toward an approaching narrow passage (instead of blindly leading to a narrow passage), passing through a narrow passage, and extending the path search more efficiently. Hence, the concept of the path planning algorithm proposed here is to design the paths in the narrow passages and those in the broad areas separately using two methodologies. The environment map *E* is illustrated in Figure 2, in which the white region is the free-moving space F and the black region is the obstacle space O. The entrance of *i*-th narrow passage is labeled with the identification point, IP*^i^*. The bidirectional rapidly exploring random trees (BiRRT) [28], which can efficiently increase the path searching efficiency, was employed to generate the path trees from the starting point and goal point at the same time. $T_{init}$ is the path tree growing from starting point $q_{init}$, $T_{goal}$ is the path tree growing from the target point $q_{goal}$. Since the area of a narrow passage is small, the optimal path of a microrobot through it is deterministic and can be efficiently designed using the well-known A* algorithm [12]. 

The environment in the broad areas, which are outside the narrow passages P, i.e., $\left\{F-P\right\}\in F$, is larger and more complex than that in the area in P. Utilizing the deterministic A* algorithm to design a path in the broad areas would be slow and inefficient. It would also be difficult to do so using the traditional stochastic RRT methodology, which uses aimless waypoint sampling. Therefore, we proposed an MIS-BiRRT based on bidirectional rapidly exploring random trees (BiRRT) [28] to efficiently obtain the path of a microrobot moving within the broad areas, which is explained in Section 3. The path growth of our MIS-BiRRT is guided to the identification point to assist the microrobot through the narrow passage. The detection of identification points and the connection of paths in the narrow passages and in the broad areas are described in the following two subsections.

### 2.1. Detection of Narrow Passages and Identification Points

**Figure 3:**
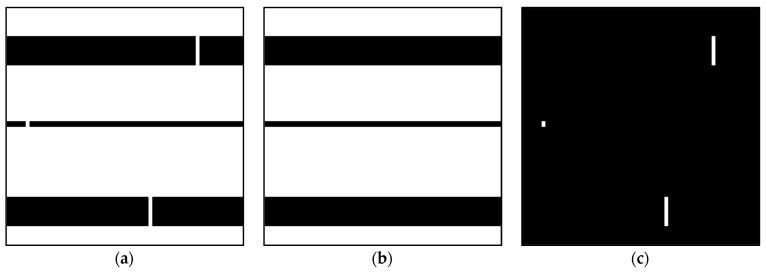
Detection of narrow passages: (**a**) environment map *E*; (**b**) opened map image $I_{o}$; and (**c**) location map of narrow passages $I_{P}$.

As illustrated in Figure 1, directly utilizing the traditional RRT algorithm to plan a path in the free-moving space F would experience difficulty trying to find a path through a narrow passage. In order to improve the efficiency of path planning, we first marked the narrow passages P in the free-moving space F. The environment map is treated as a 2D binary image for image morphology and logic operations. As demonstrated in Figure 3, the small white stripes, which are the narrow passages between the broad areas, are eroded by the image opening operation (Figure 3b). The image opening operation for the white regions employs the square structuring element with an area larger than size r of a microrobot so that the narrow passages detected later are those that the considered microrobot can pass through. Then, the location map of narrow passages $I_{P}$ can be extracted by performing the element-wise XOR logic operation between E and the opened map image $I_{o}$. $I_{o}$ could also be regarded as the broad areas $\left\{F-P\right\}\in F$.

**Figure 4:**
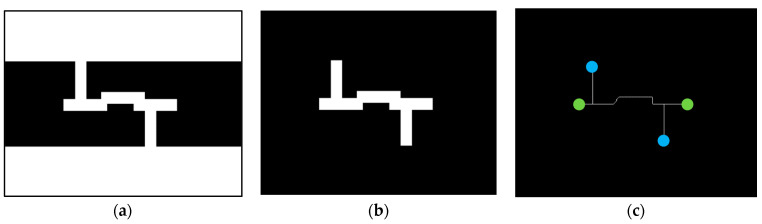
Detection of identification points in a narrow passage: (**a**) environment map E; (**b**) location map of narrow passages $I_{P}$; and (**c**) the thinning of narrow passages. The blue and green points are the feature points of the narrow passage. The blue points denote the identification points $IP_{j}^{i}$ of the narrow passage.

The white points in the location map of narrow passages $I_{P}$ yielded from morphological and logic operations are only simply independent pixels. It is still necessary to further mark the region of the narrow passage completely and label its entrance and exit. Here, the image thinning process, which can present the shape and preserve the connectivity of the adjacent white pixels, is applied to the location map of narrow passages $I_{P}$, as shown in Figure 4. As presented in Figure 4c, the endpoints of the extracted skeleton of a narrow passage are defined as the feature points of a narrow passage. However, only the feature points located around the white regions, i.e., the broad areas $\left\{F-P\right\}\in F$, in the opened map image $I_{o}$ are assigned as the identification points ${\mathrm{IP}}_{}^{i}=\left\{IP_{1}^{i},\ldots IP_{j}^{i}\ldots ,IP_{M_{i}}^{i}\right\}$ of the *i*-th narrow passage, where $M_{i}$ is the total number of the extracted identification points $IP_{j}^{i}$ of the *i*-th narrow passage. The identification points, which indicate the entrance or exit of the narrow passages, are later employed to design the path through the narrow passages.

### 2.2. Path Planning through the Narrow Passage

**Figure 5:**
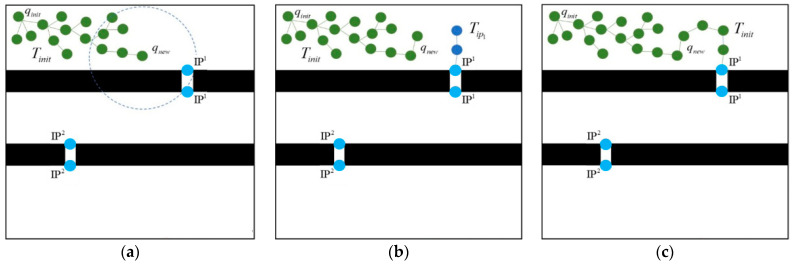
Path planning through the narrow passage: (**a**) RRT close to a narrow passage; (**b**) a new tree (blue color) growing from the identification point; and (**c**) the combined path toward the narrow passage.

The identification points of the narrow passages are to assist the RRT methodology employed in the broad areas to quickly find the narrow passages. Since the width of a narrow passage is small, this subsection also explains path planning through a narrow passage with its assigned identification points. Figure 5 illustrates an example of guiding the RRT path planning process used in the broad areas to pass through a narrow passage. As shown in Figure 5a, $q_{new}$ is the new growth waypoint generated by the RRT algorithm. When a new waypoint $q_{new}$ is within distance $d_{IP}$ from one of the identification points $IP_{j}^{i}$, the path tree $T_{init}$ should be led to the *i*-th narrow passage to construct the path of the microrobot. The closest identification point $IP_{j}^{i}$ is then assigned as a midway target point and a new path tree growth $T_{IP_{j}^{i}}$, as shown in Figure 5b, is triggered at the same time. Once the locally bidirectional RRT paths [28] between the original tree $T_{init}$ and the new tree $T_{IP_{j}^{i}}$ are connected, the new tree $T_{IP_{j}^{i}}$ is combined with the original tree $T_{init}$, as illustrated in Figure 5c. Directing the identification point in this way solves the problem faced by the traditional RRT in not finding the path to narrow passages (Figure 1). 

In addition, since the selected identification points are the entrance or exit of a narrow passage to the broad areas, the selected identification points would also be regarded as the starting and target points when planning the path through the narrow passage. As mentioned at the beginning of Section 2, the A* algorithm [12] generates good path-planning results in small and narrow regions, which can be considered a deterministic problem. The path of a microrobot passing through a narrow passage is designed between the identification points of the same narrow passage by the path planning of the A* algorithm. An identification point can be considered an interface between a broad area and a narrow passage. The path planned by the A* algorithm in a narrow passage can also be integrated into the tree $T_{init}$ (or $T_{goal}$) of the BiRRT methodology.

## 3. Multiple Importance Sampling Bidirectional Rapidly Exploring Random Trees, MIS-BiRRT

In the traditional RRT methodology [14], the direction of path growth is determined by the generation of a random guiding point, $q_{rand}$, to find the closest waypoint *q_near_* in the planned path tree. A new waypoint $q_{new}$ is then generated at a fixed distance of extension from the closest waypoint *q_near_* toward the random guiding point $q_{rand}$. However, the random guiding point $q_{rand}$ is randomly sampled on the map according to a uniform distribution. Even if the last waypoint $q_{last}$ in the path tree was close to the target point $q_{goal}$, the next waypoint $q_{new}$ could easily be far away from the target due to this aimless, random approach. 

Although traditional RRT is fast, this kind of aimless, random generation of guiding point $q_{rand}$ cannot efficiently find the path for a microrobot through complex environments and narrow passages. Hence, the multiple importance sampling strategy is designed to generate a random guiding point $q_{rand}$ such that the microrobot can be quickly led to the target point and avoid obstacles. The multiple importance sampling strategy also directs the path growth toward the narrow passages and is integrated with the BiRRT algorithm as MIS-BiRRT, jointly conducting the path planning through narrow passages, as discussed in Section 2. Moreover, the generation and selection of multiple new waypoints are proposed for MIS-BiRRT to increase the searching range to improve the efficiency of the path growth.

### 3.1. Multiple Importance Sampling

The importance function is a way to provide cues for random sampling generation. Multiple importance sampling [29] aggregates multiple importance functions to generate random samples more efficiently and in a multivariate manner, enabling it to cope with complex variation rather than just a single fixed situation. The multiple importance sampling strategy of MIS-BiRRT involves four types of importance functions, namely $Q_{goal}(q_{rand})$, $Q_{IP}(q_{rand})$, $Q_{obstacle}(q_{rand})$, and $Q_{uniform}(q_{rand})$, to sample the random guiding point *q_rand_* of path growth in a complex environment. The importance function for target $Q_{goal}(q_{rand})$ directs the path growth direction to the target point $q_{goal}$ by using the direction sampling concept [27], and it is formulated as

(1) $$Q_{goal}(q_{rand})=N(q_{rand};\;q_{goal},\;\sigma_{goal}),$$

where N(⋅) is the 2D normal distribution with the mean $q_{goal}$ and a user-defined standard deviation $\sigma_{goal}$. The importance function for identification point $Q_{IP}(q_{rand})$ navigates the path growth toward an approaching narrow passage. As mentioned in Section 2.2, the importance function $Q_{IP}(q_{rand})$ is adopted when the last growth waypoint $q_{last}$ in the path tree is close to an identification point $IP_{j}^{i}$ of the *i*-th narrow passage, i.e., $\left|q_{last}-IP_{j}^{i}\right|\leq d_{IP}$. The importance function for identification point $Q_{IP}(q_{rand})$ is defined as

(2) $$Q_{IP}(q_{rand})=N(q_{rand};\;IP_{j}^{i},\;\sigma_{IP}),$$

which is a normal distribution with the closest identification point $IP_{j}^{i}$ as the mean and the standard deviation $\sigma_{IP}$.

**Figure 6:**
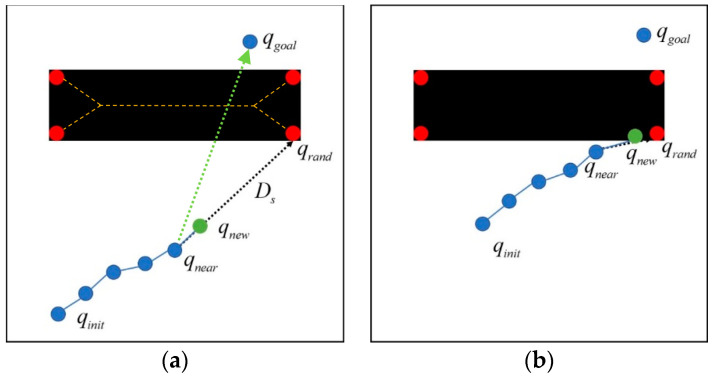
The importance function of obstacle avoidance: (**a**) navigating the microrobot across the obstacle and (**b**) when the path is too close to the obstacle, the new waypoint may clash with it when drawing from this importance function.

If there is an obstacle in front of the guiding direction of the previous two importance functions, the importance function of obstacle avoidance $Q_{obstacle}(q_{rand})$ is then employed to design the path growth across the obstacle [27]. We applied the image skeletonization process to the obstacle regions O with black pixels in the 2D binary image of environment map E. As shown in Figure 6a, the orange dashed line is the extracted skeleton of an obstacle region, and the red points are the $H_{k}$ endpoints $FP_{h}^{k},\;h=1,\ldots ,H_{k}$ of the *k*-th obstacle that blocks the guiding direction to the target point (green dotted line). The endpoint $FP_{h}^{k}$ of the obstacle skeleton, which is closest to the path tree, was selected as the random guiding point $q_{rand}$ to navigate the microrobot across the obstacle. The importance function of obstacle avoidance $Q_{obstacle}(q_{rand})$ is denoted as

(3) $$Q_{obstacle}(q_{rand})=N(q_{rand};\;FP_{h}^{k},\;\sigma_{obstacle}),$$

which is a normal distribution with the closest obstacle endpoint $FP_{h}^{k}$ as the mean and the standard deviation $\sigma_{obstacle}$. From practical implementation, however, it is noticed that once the path is very close to the obstacle, as shown in Figure 6b, the new waypoint $q_{new}$ generated from importance function $Q_{obstacle}(q_{rand})$ repeatedly clashes with the obstacle such that the RRT algorithm is stuck here. Hence, once the new waypoint $q_{new}$ sampled from the importance function $Q_{obstacle}(q_{rand})$ is located in the obstacle region O, the new waypoint $q_{new}$ needs to be resampled from another importance function $Q_{uniform}(q_{rand})$:

(4) $$Q_{uniform}(q_{rand})=U(q_{rand};\;F),$$

where U(⋅) is the 2D uniform distribution. This draws the random guiding point $q_{rand}$ within the free-moving space F uniformly. By using the importance function $Q_{uniform}(q_{rand})$, the new path growth waypoint $q_{new}$ has a high probability of being able to escape from its trap and search for another feasible direction of movement. The mechanism of the importance function of uniform resampling $Q_{uniform}(q_{rand})$ is designed by referring to many stochastic optimizations [30], which can overcome the local solutions.

### 3.2. Generation of Multiple New Waypoints

In order to increase the searching range of the RRT methodology, Multi-RRT [26] draws several new waypoints for path growth simultaneously. However, these growth points are all at fixed angle intervals, and so many of them are scattered blindly and are not used efficiently. Here, the concept of multiple new waypoint generation is incorporated into our MIS-BiRRT, which proposes a more efficient mechanism to generate and select new waypoint samples in order to increase the path-searching range of a microrobot.

**Figure 7:**
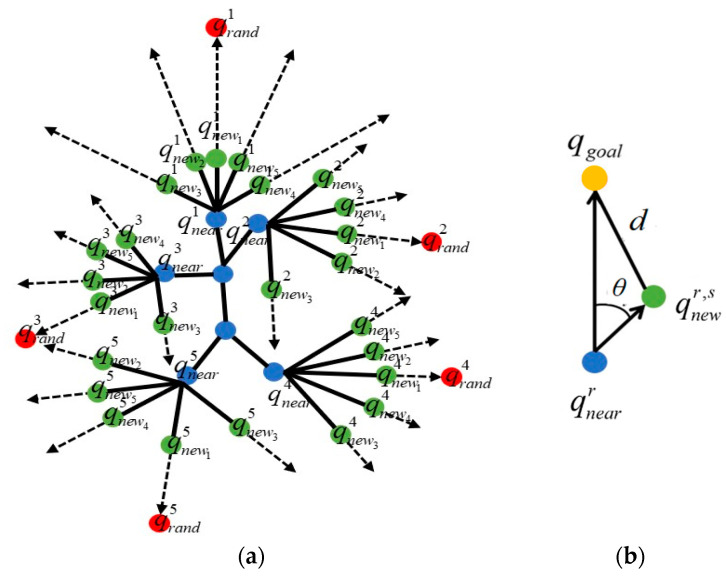
Generation of multiple new waypoints. (**a**) M=5. (**b**) Evaluation of performance index $p(q_{new}^{r,s})$. The blue dots are the waypoints on the MIS-BiRRT tree, the red points are the random guiding points $q_{rand}^{r}$, and the green dots are the generated new waypoints $q_{new}^{r,s}$ from the waypoint $q_{near}^{r}$ of the path tree.

It was explained in the previous subsection how to determine the path growth of a new waypoint $q_{new}$ according to the sampling of random guiding point $q_{rand}^{}$. In the multiple new waypoints generation methodology of MIS-BiRRT, there are M random guiding points $q_{rand}^{r},\;r=1,\ldots ,M$ sampled from each of the four importance functions, $Q_{goal}(q_{rand})$, $Q_{IP}(q_{rand})$, $Q_{obstacle}(q_{rand})$, and $Q_{uniform}(q_{rand})$. Each selected importance function corresponding to a random guiding point $q_{rand}^{r}$ draws M new waypoints $q_{new}^{r,s},\;s=1,\ldots ,M$ from the corresponding closest waypoints $q_{rand}^{r}$. In order to maintain the smooth movement of the microrobot, the variation in orientation angle between each step, i.e., between $q_{new}^{r,s}$ and $q_{near}^{r}$, is limited within a feasible range. As illustrated in Figure 7a, there are $M^{2}$ new waypoints $q_{new}^{r,s},\;r=1,\ldots ,M,\;s=1,\ldots ,M$ used to search the path of a microrobot at one time. In order to maintain a fixed computational load, only M of these $M^{2}$ new waypoints are actually selected to extend the paths. The performance index of each waypoint $q_{new}^{r,s}$ is estimated by the possibility of path growth toward the target point:

(5) $$p(q_{new}^{r,s})=\alpha d(\vec{q_{near}^{r}q_{new}^{r,s}},\;\vec{q_{near}^{r}q_{goal}})+\beta \theta (\vec{q_{near}^{r}q_{new}^{r,s}},\;\vec{q_{near}^{r}q_{goal}}),$$

where α and β are the weight constants and $d(\vec{q_{near}^{r}q_{new}^{r,s}},\;\vec{q_{near}^{r}q_{goal}})$ and $\theta (\vec{q_{near}^{r}q_{new}^{r,s}},\;\vec{q_{near}^{r}q_{goal}})$ evaluate the distance and angle, respectively, between the vector of the hypothesized path growth and the vector toward the target point, as shown in Figure 7b. The vector $\vec{q_{near}^{r}q_{goal}}$ denotes the direction and distance from the waypoint $q_{near}^{r}$ of the path tree to the target point, and the vector $\vec{q_{near}^{r}q_{new}^{r,s}}$ implies the extension of path growth from the waypoint $q_{near}^{r}$ to a hypothesized new waypoint $q_{new}^{r,s}$. The  new waypoints with the smallest values of $p(q_{new}^{r,s})$ are selected to generate the new waypoints, and the others are excluded. The purpose of growing multiple new waypoints each time is to increase the searching range and the probability of path growth toward the target point. Furthermore, the smaller value of $p(q_{new}^{r,s})$ in (5) implies that there is a smaller difference in the orientation angle between the selected new waypoint $q_{new}^{r,s}$ and the target point. This selecting mechanism can also ensure that the planned path is smooth toward the target point.

When designing the path tree $T_{goal}$ of MIS-BiRRT generated from the target point $q_{goal}$ toward the starting point $q_{init}$, the starting point $q_{init}$ is regarded as the target during multiple importance sampling and the generation of the multiple new waypoints mentioned above. Once the bidirectional path trees $T_{init}$ and $T_{goal}$ of MIS-BiRRT have at last approached within a predefined (small) distance, the collision-free path τ for the microrobot through the complex environment is obtained by connecting the bidirectional path trees. The planned path τ is then defined from the connected waypoints and the corresponding parent waypoints in the bidirectional path trees $T_{init}$ and $T_{goal}$.

## 4. Simulation and Analysis

**Figure 8:**
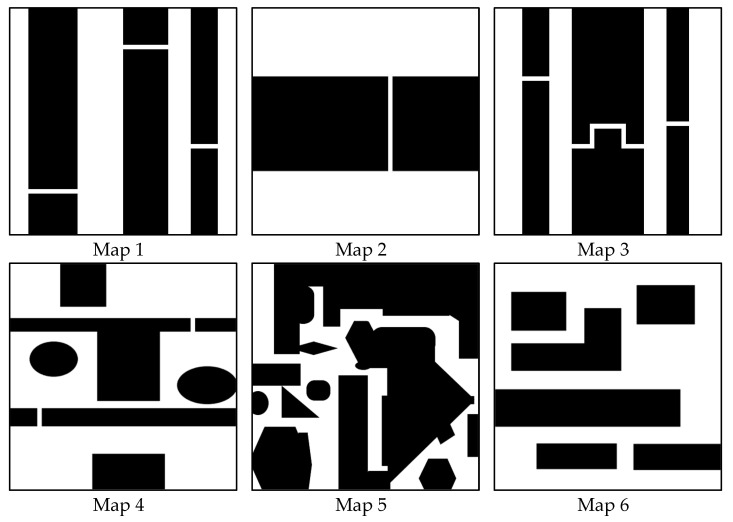
Environmental maps.

We illustrate six environmental maps, as shown in Figure 8, to test the path planning efficiency of the proposed MIS-BiRRT methodology. Maps 1–5 have narrow passages, but Map 6 does not, and each map is 2.4 m × 2.4 m. Map 5 is a complex environment map with many obstacles and narrow passages. The starting point in each map is set as the left top corner of the environment, and the goal point in each map is set at the right bottom corner. The considered microrobot is a legged one [2] with a size of 40 mm × 40 mm. In the simulations of Section 4.1, the proposed MIS-BiRRT algorithm is compared with other path planning algorithms, namely RRT [11], BiRRT [28], RRT* [31], PRM [13], A* [12], and hybrid A* [32], to demonstrate the superiority of the method described in this paper. Then, the ablation studies, which simulate the path planning by removing certain components of the proposed MIS-BiRRT, is provided in Section 4.2. The impact of these components in MIS-BiRRT could be measured from the results of ablation studies.

Quantitative indicators, including the computational time (time) and the total length (length) of the planned path, are used to evaluate the path planning algorithms in the following simulation results. The better a path planning algorithm is, the shorter its computational time and the shorter the planned path it can generate for the microrobot through the environment. The total number of new sampled waypoints (total waypoints) and successfully extended new waypoints (successful waypoints) are also recorded as quantitative indicators for evaluating the algorithms. Since the new sampled waypoints could clash with obstacles and have to be resampled repeatedly, the successfully extended ratio (success ratio) is defined to calculate the ratio of successful waypoints to total waypoints. An efficient sampling mechanism in an RRT-type methodology should have a higher success ratio, which indicates that a smaller number of new sampled waypoints fail to grow the path.

### 4.1. Simulation Results and Comparison

The simulation results of the proposed MIS-BiRRT compared with the RRT, BiRRT, RRT*, PRM, A*, and hybrid A* algorithms are presented in Table 1. The stochastic algorithms, RRT, BiRRT, RRT*, MIS-BiRRT, and PRM, were evaluated by taking the average of thirty executions of each algorithm. Since PRM randomly draws the nodes in the entire environment and connects the adjacent nodes as the planned path, the path planned by PRM is the longest of all methods. The RRT* method, which is an asymptotically optimal modification of RRT, is the fastest of all methods considered here. However, since RRT* omits a more extensive search by extending the tree, the path length obtained by RRT* is the longest among the category of RRT algorithms. Compared with RRT and BiRRT, MIS-BiRRT is superior in terms of path length, total waypoints, and success ratio. Since the proposed MIS-BiRRT can mark the narrow passages, quickly find the path through them, and generate multiple import waypoints over a wide area, it can effectively reduce unnecessary path searches. Because MIS-BiRRT utilizes image processing and generates more sampling waypoints, however, it requires more computation to generate, resulting in a slightly higher computational time than that required by RRT and BiRRT.

In terms of path length, the hybrid A* algorithm, a deterministic algorithm, is the best and can be regarded as the optimal path solution in each environment. Because the solution to the problem is explicit, the A* and the hybrid A* algorithms yield shorter paths than the stochastic algorithms. However, the A* algorithm requires an enormous amount of calculation and the computational time is considerably large, which can be seen from the comparison. The overall efficiency of the A* algorithm is not high. Except in Map 5, the hybrid A* algorithm takes even less time than some RRT-type methods. However, in a complex environment such as Map 5, the hybrid A* algorithm is obviously slower than all RRT-type methods.

Next, we discussed the simulation differences of each method in these six maps. Maps 1–4 all contain significant narrow passages in the environments. Map 2 has a long and straight, narrow passage, and Map 3 has a crooked narrow passage. Map 4 looks like two rooms with narrow passages as the entrances. Since there are thin and long narrow passages in Map 1–4, the path lengths in Map 1–4 obtained by the proposed MIS-BiRRT algorithm are close to those obtained by the A* or the hybrid A* algorithm. This is due to the fact that a deterministic algorithm can easily yield the optimal solution in a narrow space. In contrast, in Map 5, with several short narrow passages, or in Map 6, with no narrow passages, where the RRT methodologies would have stochastic results in the broad areas, the path length planned by the proposed MIS-BiRRT algorithm is close to that obtained by RRT, BiRRT, or RRT*. In addition, since there are multiple long narrow passages in Map 1, 3, or 4, which would be multiple difficulties to block the waypoints sampling, the computational time spent by RRT* algorithm in Map 1, 3, or 4 is longer than that in Map 2, 5, or 6. Due to the proposed MIS-BiRRT could adaptively switch between the stochastic and deterministic manners, the computational time taken by our method does not vary much in different environments. Since there are no narrow passages at all in Map 6, which would not block the waypoints sampling of RRT-type methodology, the success ratio of RRT, BiRRT, or RRT* in Map 6 is higher than that in Map 1 to 5.

**Figure 9:**
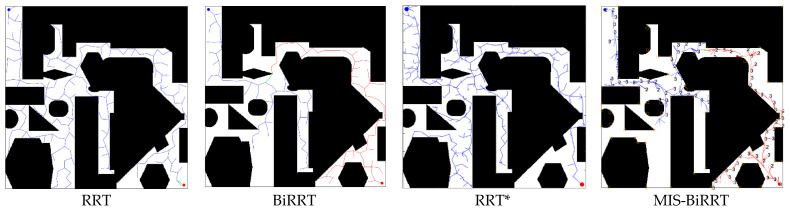

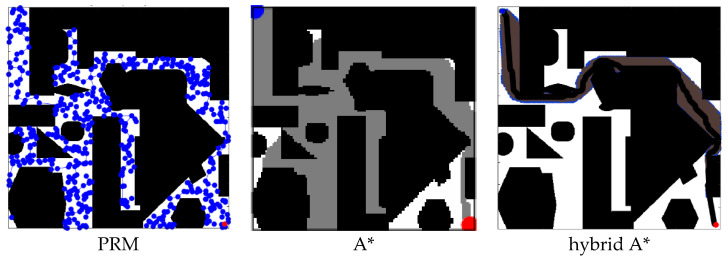
Comparison of searching range in Map 5.

**Figure 10:**
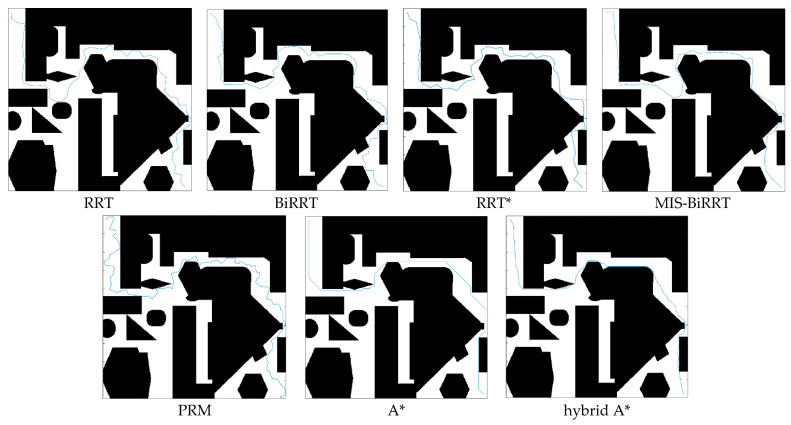
Comparison of planned path in Map 5.

Then, we visualized and compared the results of path planning for Map 5. Figure 9 and Figure 10 present the search ranges and the resulting paths, respectively. In Figure 9, the blue point at the left top corner is the starting point, and the red point at the right bottom corner is the goal point. PRM randomly and chaotically draws the nodes in the entire environment, which displays the characteristic of stochastic methodology with clueless sampling (blue dots). As shown in Figure 9, RRT needs to sample an enormous number of waypoints, and BiRRT is aimlessly searching with many waypoints even though it combines searching from another direction. RRT* searches the environment with less tree extension than RRT or RRT* such that its resulting path is longer than that of RRT or RRT*. Due to the characteristics of multiple importance sampling designed in the proposed MIS-BiRRT, there are clues for generating the waypoints such that unnecessary searching could be reduced. By contrast, the A* and hybrid A* algorithm plans the path by searching regions of the map, indicated by gray in Figure 9, and so they would have taken more time than the stochastic methodologies. The A* or the hybrid A* algorithm shows the straight and shortest planned path in Figure 10, but RRT, BiRRT, and RRT* paths are tortuous. The path planned by MIS-BiRRT shows the features of both deterministic and stochastic algorithms as it adapts throughout the process.

### 4.2. Ablation Studies

For the following simulations, we tried to remove certain modules of the proposed MIS-BiRRT methodology to see the effectiveness of each (see Table 2). The BiRRT algorithm [22], which is the infrastructure of the MIS-BiRRT methodology, provides the importance function $Q_{goal}(q_{rand})$ for directing the waypoints toward the goal point, i.e., BiRRT + G in Table 2. The BiRRT + GO method in Table 2 denotes the BiRRT algorithm accompanied by the importance functions $Q_{goal}(q_{rand})$ and $Q_{obstacle}(q_{rand})$ that guide the path growth toward the target point and avoid obstacles. In addition, the Multi-RRT [20] concept is implemented to modify the BiRRT + GO method accompanied with the importance function $Q_{uniform}(q_{rand})$ (Multi-BiRRT in Table 2), which simultaneously generates five new waypoints spaced at a fixed angle toward a guiding point *q_rand_*.

BiRRT + G, which employs Goal Bias [20] as a simple clue for guiding the path growth toward the goal point, is the fastest algorithm presented in Table 2 but also generates the longest path length. The BiRRT + GO method has one more importance function than BiRRT + G, which refers to direction sampling [21] for obstacle avoidance; however, the guidance of these two importance functions in the BiRRT + GO algorithm does not generate good planning results and even takes considerable time searching for a path. This inefficiency of the BiRRT + GO method is due to the poor integration of the importance functions, which simply want to move toward the target point and avoid obstacles, and do not provide any leading clues to or through the narrow passages. Both the BiRRT + G and BiRRT + GO methods waste many samples searching for a feasible path, and, as such, they have a lower success ratio (Table 2). BiRRT + G or BiRRT + GO spent less computational time on path planning in Map 6, which contains no narrow passages, than in other environmental maps.

In contrast, Multi-BiRRT generates multiple new waypoints to increase its path-searching hypotheses compared with the BiRRT + G and BiRRT + GO methods. Since more samples are drawn from the importance functions, the diversity of sampled waypoints is increased, and the searched space is wider, causing most of the Multi-BiRRT length results to be shorter than those of the BiRRT + G and BiRRT + GO methods. We also can see that Multi-BiRRT spent less computational time to obtain the path in Maps 1, 2, 3, and 5 than in Map 4 or 6, in which the path is not so obvious to be obtained. Since Map 5 only contains several short narrow passages, and Map 6 has no narrow passages, due to the efficient multiple important sampling of the proposed MIS-BiRRT, the path length of MIS-BiRRT is much better than that of the other algorithm in Map 5 or 6.

**Figure 11:**
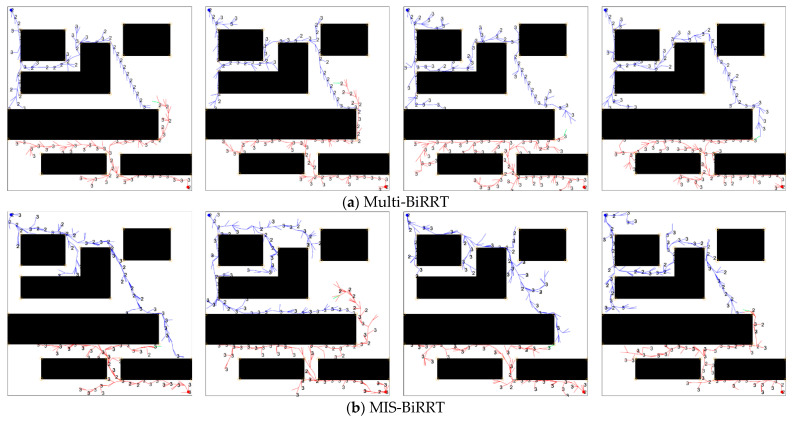
Comparison of path trees grown by (**a**) Multi-BiRRT and (**b**) MIS-BiRRT. The number 2 and 3 indicate the waypoint generated from the importance function $Q_{obstacle}(q_{rand})$ and $Q_{uniform}(q_{rand})$, respectively.

Compared with the proposed MIS-BiRRT, however, the diversity of Multi-BiRRT is still insufficient. Figure 11 illustrates the four path-growing processes of Multi-BiRRT and MIS-BiRRT in Map 6. We can see that the shapes of the path trees grown by the Multi-BiRRT method are very similar. Sampling schemes with importance functions represent a trade-off between degeneracy and impoverishment [24]. The degeneracy phenomenon is due to aimless sampling, similar to that in the traditional RRT algorithm, whereas impoverishment can be regarded as the overconcentration of the samples drawn from the importance function, such as in BiRRT + G or BiRRT + GO, and it may cause the hypotheses to center only on a local solution. In the proposed MIS-BiRRT methodology, multiple new waypoints are sampled from multiple importance functions and then selected according to an evaluation of the sampled waypoints. The MIS-BiRRT algorithm can generate various path tree shapes, as shown in Figure 11b. The proposed MIS-BiRRT methodology utilizes the importance functions as clues to concentrate waypoint sampling on high-probability areas while preserving randomness by using multiple various samples. Although the proposed MIS-BiRRT requires more computational time than BiRRT + G, its path length, total waypoints, and success ratio results are the best, which proves that the proposed MIS-BiRRT can improve the efficiency of path planning for microrobots through complex environments with narrow passages.

## 5. Conclusions

Due to its extremely small size, a microrobot is especially suitable for performing tasks in narrow spaces. This paper proposed the MIS-BiRRT path planning algorithm to efficiently navigate a microrobot through a complex environment with narrow passages. Unlike general RRT algorithms that use purely stochastic methods for path planning, MIS-BiRRT adopts both the stochastic and deterministic methods during path planning, utilizing the advantages of each. The narrow passages are detected by image processing, and the identification points are labeled to denote the entrance or exit of a narrow passage. Once the MIS-BiRRT path growth is connected to an identification point, the A* algorithm is then utilized to quickly find the optimal path through the narrow passage. After exiting the narrow passage, the path planning switches back to the MIS-BiRRT methodology such that the inefficiency of the stochastic algorithm in planning a path through narrow passages can be reduced. In order to increase the sampling efficiency of the RRT path growth in the broad space, the multiple importance sampling mechanism of MIS-BiRRT is designed to integrate several importance functions to guide the path growth toward the target point, ultimately connecting to the identification point of a narrow passage and avoiding any obstacles. Multiple waypoint sampling and selection are also incorporated into MIS-BiRRT to increase the variance of generated waypoints while reducing useless ones. Through simulation and comparison, it is proved that the proposed MIS-BiRRT improves the overall performance of microrobot path planning in complex environments.

Since the proposed MIS-BiRRT involves more calculations for image processing, multiple waypoints generation and selection, and the integration of multiple samples, the computational time is longer than for simple RRT methodologies. It could be reduced by being implemented via low-level programming libraries when practical. In the future, the proposed algorithm can be applied in microrobots with microprocessors or embedded computation systems. The proposed path-planning method will also be further revised for practical applications by considering the dynamics of the microrobots.

## Figures and Tables

**Table 1:** Comparison of path planning results.

Environment	Algorithm	Time (s)	Length (m)	Total Waypoints	Successful Waypoints	Success Ratio
Map 1	RRT	5.998	7.739	10,221	260	0.025
	BiRRT	4.258	7.875	4884	186	0.036
	RRT*	2.839	8.113	7360	1935	0.263
	MIS-BiRRT	12.528	7.119	783	242	0.309
	A*	420.536	6.905	-	-	-
	Hybrid A*	3.038	6.840	-	-	-
	PRM	5.466	9.642	-	-	-
Map 2	RRT	6.051	4.265	4028	263	0.065
	BiRRT	3.423	4.266	1907	152	0.080
	RRT*	0.490	4.321	1608	547	0.340
	MIS-BiRRT	10.231	3.741	610	254	0.416
	A*	183.623	3.758	-	-	-
	Hybrid A*	1.666	3.684	-	-	-
	PRM	5.347	6.202	-	-	-
Map 3	RRT	5.878	5.278	8399	257	0.031
	BiRRT	6.001	5.292	9658	255	0.026
	RRT*	3.817	5.452	9830	2299	0.234
	MIS-BiRRT	12.933	4.791	332	120	0.361
	A*	6025.050	4.720	-	-	-
	Hybrid A*	4.580	4.585	-	-	-
	PRM	98.856	7.378	-	-	-
Map 4	RRT	7.569	8.011	5482	321	0.059
	BiRRT	6.321	8.297	6263	266	0.042
	RRT*	3.112	8.247	5981	2102	0.351
	MIS-BiRRT	9.634	7.139	1052	419	0.398
	A*	634.071	6.778	-	-	-
	Hybrid A*	5.831	6.720	-	-	-
	PRM	5.607	10.787	-	-	-
Map 5	RRT	5.948	5.770	6384	198	0.031
	BiRRT	3.464	5.728	3731	115	0.031
	RRT*	1.833	5.846	5048	1343	0.266
	MIS-BiRRT	10.425	5.576	2686	306	0.114
	A*	230.075	4.819	-	-	-
	Hybrid A*	12.050	4.800	-	-	-
	PRM	46.158	7.694	-	-	-
Map 6	RRT	7.184	5.756	2294	314	0.137
	BiRRT	3.054	5.620	1338	138	0.103
	RRT*	0.436	5.916	1440	729	0.506
	MIS-BiRRT	9.816	5.287	940	339	0.361
	A*	5911.466	4.748	-	-	-
	Hybrid A*	4.234	4.676	-	-	-
	PRM	5.653	11.337	-	-	-

**Table 2:** Comparison of ablation studies.

Environment	Algorithm	Time (s)	Length (m)	Total Waypoints	Successful Waypoints	Success Ratio
Map 1	BiRRT + G	4.300	7.861	9346	188	0.020
	BiRRT + GO	31.724	7.748	8819	178	0.020
	Multi-BiRRT	14.864	7.273	913	116	0.127
	MIS-BiRRT	12.528	7.119	783	242	0.309
Map 2	BiRRT + G	3.801	4.195	4235	163	0.038
	BiRRT + GO	34.964	4.01	2528	120	0.047
	Multi-BiRRT	17.874	3.895	262	96	0.366
	MIS-BiRRT	10.231	3.741	610	254	0.416
Map 3	BiRRT + G	5.764	5.102	12,793	237	0.019
	BiRRT + GO	58.282	5.222	12,888	242	0.019
	Multi-BiRRT	15.499	4.908	264	65	0.246
	MIS-BiRRT	12.933	4.791	332	120	0.361
Map 4	BiRRT + G	6.184	8.236	8010	260	0.032
	BiRRT + GO	49.765	8.044	6517	240	0.037
	Multi-BiRRT	25.821	7.430	1222	175	0.143
	MIS-BiRRT	9.634	7.139	1052	419	0.398
Map 5	BiRRT + G	6.858	6.172	4386	77	0.018
	BiRRT + GD	33.496	6.294	3542	77	0.021
	Multi-BiRRT	14.948	5.989	1682	134	0.080
	MIS-BiRRT	10.425	5.576	2686	306	0.114
Map 6	BiRRT + G	2.981	5.602	1846	134	0.073
	BiRRT + GO	20.174	5.559	1769	132	0.075
	Multi-BiRRT	25.484	5.873	2356	134	0.057
	MIS-BiRRT	9.816	5.287	940	339	0.361

## Data Availability

Not applicable.
